# Enhancing the Accuracy of Linear Finite Element Models of Vehicle Structures Considering Spot-Welded Flanges

**DOI:** 10.3390/ma14206075

**Published:** 2021-10-14

**Authors:** Luis Martins, Gregorio Romero, Berta Suarez

**Affiliations:** Mechanical Engineering Department, Escuela Técnica Superior de Ingenieros Industriales, Universidad Politécnica de Madrid, C/José Gutiérrez Abascal, 2, 28006 Madrid, Spain; gregorio.romero@upm.es (G.R.); b.suarez@upm.es (B.S.)

**Keywords:** finite-element model, frequency response function, vibration testing, vehicle structures, correlation analyses

## Abstract

Structural engineering simulations have required increasingly complex computational models to replace physical tests accurately. This work focuses on the numerical analysis of vehicle body structures, whose size and complexity make the use of very accurate nonlinear models unfeasible due to the prohibitive computational costs involved. The purpose of this study is to find a new approach to model spot-welded joints in linear finite element models of thin-wall vehicle body structures, improving the accuracy of the model without increasing its complexity. Using a set of simplified nonlinear models, we fitted the stiffness and damping properties of these welded joints and used those adjusted values into a linear model of the entire vehicle body structure. The results were compared with experimental tests, showing a clear improvement in the accuracy of the modal and frequency responses provided by the linear finite element model, but keeping its initial complexity level. The adjusted model was then used in an optimization analysis to reduce the structure’s weight, leading to interesting cost savings and important reductions in the use of natural resources and carbon emissions.

## 1. Introduction

The level of correlation between the behaviour of a physical system and the results obtained from a finite element (FE) model is essential in many engineering areas. The accuracy of results from finite element analysis (FEA) increases with the correlation levels, thus reducing the demand for prototypes and providing high savings in time and resources for engineering projects. This statement is especially relevant in the automotive industry [1,2,3], where the use of FE models is necessary and determinant to accelerate product development cycles and increase the cost and time efficiency of new vehicle projects.

From this perspective, the validation of analytical simulation models became a decisive factor in developing new products, especially since the use of advanced computational resources became economically viable. In 1992, Baker [4] compared different methods to correlate analysis predictions with test data, including modal matrices and comparisons of frequency response functions. In 1999, Brughmans et al. [5] studied FEA model updating techniques for correlation improvement in order to support the implementation of a state-of-the-art NVH CAE design optimization environment. In 2005 Schedlinski et al. [6] presented an FEA model update technique based on a sheet metal gauge update. They used the Modal Assurance Criterion (MAC) to compare the modal consistency of the original and updated models. Splendi et al. [7] presented in 2013 a technique to tune the behaviour of damping patches in FE models to improve their correlation with experimental tests. They used the MAC to verify the modal consistency and compared the point mobility FRF main peak amplitudes between the experimental and numerical results. In 2017, Rotondella et al. [8] proposed a novel welding representation for metallic joints, using the MAC to measure the numerical–experimental correlation and validate the newly proposed modelling technique.

Despite this continuous improvement of FEA [2,9,10,11,12], the accuracy level of linear models remains limited by the lack of nonlinear phenomena representation, such as friction and contact reaction forces. In addition, the complexity of nonlinear models needed to represent those phenomena limits its use to specific boundary conditions. Otherwise, it would be unfeasible to solve them considering the computational resources currently available. For this reason, linear models are traditionally mainly used in these situations.

In the case of a vehicle body subjected to a vibrational analysis, the structure is typically composed of a set of thin-wall stamped steel sheets connected by spot welds at their edges. As a consequence of these constructive properties, nonlinear contact between flanges of adjacent sheets will always occur. Considering the high complexity given by the deep detail level required, we decided to build and analyse the simulation models using the FE technique, taking advantage of its flexibility in design representation and its ability to simulate distinct mechanical elements. On the other hand, to perform vibration analyses, we should analyse these FE models using the eigenvalue approach [13,14] at least up to 100 Hz, to capture the highest energy modes of the structure, leading to an undesirable combination of a large nonlinear model with a very demanding computational effort.

In this work, we aim to present a new modelling methodology to increase the accuracy of linear FEA, thus avoiding exceeding the computational resources currently available. To this end, we compared the correlation levels obtained with both linear and nonlinear models of the whole vehicle structure.

**Figure 1:**
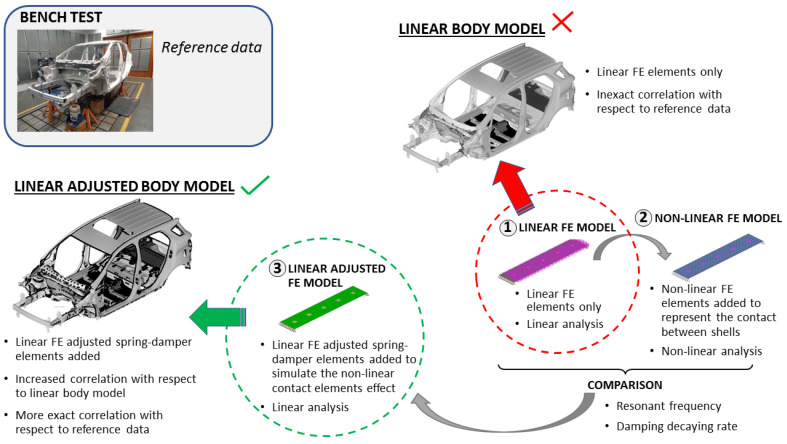
Scheme followed to develop the adjusted linear FE model of the vehicle body structure.

Taking a vehicle body structure, we performed a set of bench tests to capture its modal behaviour and its frequency response functions (FRFs). We also built a reference FE model of the same vehicle body structure to simulate an identical analysis to the field tests performed on the bench test (Figure 1). We compared the results obtained from the reference FE model of the body structure with those obtained from the bench tests and computed the correlation coefficients. 

After that, we built three FE test models to represent a small part of the structure welded flange. We first built an FE test model using only linear parameters, thus avoiding nonlinear behaviours in the contact area between the flanges of adjacent panels. We then built a second nonlinear FE test model using the first model as a base, adding to the first model the nonlinear contacts of the flanged areas and computed the difference of behaviour between both models. We also built a third linear FE test model using the first model as a base but now adding linear spring-damper elements to simulate the nonlinear behaviour at the contact areas. The elastic constants of these spring-damper elements were adjusted using the differences found between the responses of the first and second FE models.

We finally built an adjusted FE model of the whole vehicle body structure, adding to the reference FE model of the body structure the linear spring-damper elements previously adjusted with the help of the FE test models. We then performed with this adjusted model the same analyses carried out with the bench test and the reference FE model, comparing the adjusted model results with the bench test results, and computing the correlation coefficients.

The results of the adjusted FE model of the body structure showed an improvement of 10.6% in the correlation coefficient, compared to the results of the initial reference model. The modal behaviour coefficients and the visual comparison of the FRF curves also show a higher correlation given by the adjusted model, confirming the validation of the adjustment methodology proposed here.

During the state-of-the-art review, we detected a knowledge gap regarding the representation of nonlinear contacts in large finite element models subjected to frequency domain analysis. To overcome this gap, the objective of this work is to find a new approach to model spot-welded flanged joints into linear finite element models of thin-wall vehicle body structures, using a nonlinear model behaviour as a reference. In this way, we could improve the accuracy of the modal and frequency response function results without increasing the complexity of the FE model analysis.

## 2. Materials and Methods

### 2.1. Tests Performed on the Bench Test. Reference Data

#### 2.1.1. Test Definition

**Figure 2:**
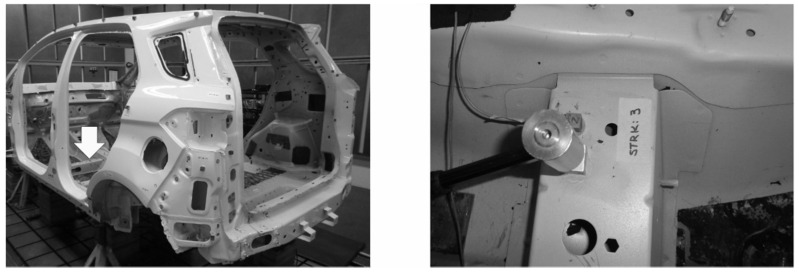
Experimental test body (**left**) and impact hammer load on the front seat attachment (**right**).

Based on the vehicle manufacturer’s experience, we performed an experimental modal analysis of the physical structure of the vehicle body on a bench test (Figure 2, left), as explained in the following paragraphs. From this test, we generated a reference set of results to support the correlation analysis of the FE models.

We isolated the vehicle structure from the other parts of the vehicle and supported it on a set of four air springs. Then, we distributed 152 triaxial accelerometers on the structural members and large panels, forming a mesh.

We used two electrodynamic shakers (Tira GmbH, Schalkau, Germany) to excite the body structure with a time-domain burst random noise. We also configured the shape of the power spectral density (PSD) of the input signal to concentrate the higher amount of energy within the frequency range from 10 to 60 Hz, where we can usually find the main eigenmodes.

We acquired the response of the vehicle body structure through a mesh of accelerometers (PCB Piezotronics, Depew, NY, USA). We identified the main body structure eigenmodes and gathered their eigenfrequencies and damping ratios. We performed three sequential runs with fifty sample records per run to cover all excitation and response points. In this way, the data analyses were more accurate and faster. We then processed these data to compute the eigenfrequencies and eigenmodes of the vehicle body structure.

We also used an impact hammer (PCB Piezotronics, Depew, NY, USA) to perform an impact test by exciting, one at a time, the four shock tower attachment points of the vehicle body structure. We applied a 1.0 N pulse at each point on the x, y, and z directions (dir) separately. We measured the magnitude and phase of the accelerations at the driver seat attachment point (Figure 2, right) in the x, y and z-dir.

We performed an average of 10 impacts per direction to smooth the transfer functions and improve data accuracy even more. We computed the fast Fourier transform (FFT) to convert the acquired data from the time to frequency domains. In this way, we obtained the frequency response function (FRF) for each point and direction.

In total, we acquired 72 FRF data sets (4 excitation points × 3 excitation directions × 3 response point directions × 2 complex roots—magnitude and phase). Although we used the entire data set for the calculations, we just plotted the amplitude of the root mean square (RMS) of the four excitation points to reduce the number of graphics shown.

#### 2.1.2. Eigenfrequencies

Table 1 gathers the eigenfrequencies and damping ratios of the main eigenmodes of the body structure found in the bench test, from 10 to 60 Hz.

### 2.2. Reference FE Model of the Vehicle Body Structure

#### 2.2.1. Model Definition

**Figure 3:**
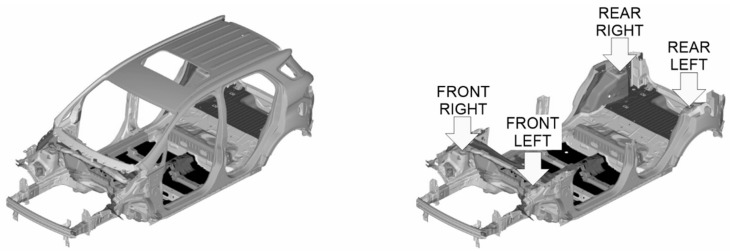
Reference FE model of the vehicle body structure (**left**) and body structure excitation points (**right**).

We built a reference FE model of the vehicle body structure (Figure 3, left). We used the Hyperworks software pack to support the FE model building (Hypermesh), the FE analysis solving (Optistruct), the post-processing FRF graphics (Hypergraphics), visualization (Hyperview), MAC (Hyperview NVH module) and FRAC (Compose) calculations. We also used MS Excel to plot additional graphics other than FRFs.

The FE model was meshed based on the vehicle manufacturer’s experience, as summarized below. We used a 5 mm meshing size with a minimum size of 2 mm for refinement at complex design regions like the edges, flanges, beads, and attachment points. We used TRIA3 and QUAD4 2D shell elements to mesh stamped parts such as panels and structural beams; HEXA8 3D + RBE3 1D elements to represent spot welds and adhesive connections; RBE2 1D elements to represent bolts and nuts, and CBUSH 1D spring-damper elements for the adjusted model tuning. We used linear material properties to represent the stamped parts and the welds, made of steel (Young’s modulus = 210.0 GPa, Poisson’s ratio = 0.30 and density = 7900 Kg/m^3^), as well as the polymeric adhesive material (Young’s modulus = 1.9 GPa, Poisson’s ratio = 0.42 and density = 1190 Kg/m^3^).

We performed a free-free modal analysis (Normal Modes solver solution) with the reference FE model to get the same mode shapes [15] as those obtained in the bench test. We also simulated the hammer impact test (Frequency Response Modal solver solution) using a frequency response analysis to find the FRFs from the four shock tower attachments and considering the six degrees of freedom [16] (Figure 3, right), like in the bench test. We extracted the eigenvalues using the Lanczos’ method, considering a structural critical damping ratio of 0.008 [17,18] from 0 to 100 Hz.

We used the Modal Assurance Criterion (MAC) and the Frequency Response Assurance Criterion (FRAC) metrics to measure the differences between the results obtained with the bench test and the reference FE model.

The MAC [19,20,21] is a normalized single-value metric that estimates the consistency between eigenvectors from different sources. We computed it to assess the accuracy of the modal behaviour of the reference FE model. We contrasted the eigenvectors found from the reference FE model with those found in the bench test, focusing on the frequency range of concern, from 10 to 60 Hz. We computed the MAC as follows [21]:

(1) $${\mathrm{MAC}}_{\left(a,x\right)}=\frac{\left|\sum_{j=1}^{N_{f}}{\left\{\varphi_{a}\right\}}_{j}{\left\{\varphi_{x}\right\}}_{j}\right|}{\left(\sum_{j=1}^{N_{f}}{\left\{\varphi_{a}\right\}}_{j}^{2}\right)\left(\sum_{j=1}^{N_{f}}{\left\{\varphi_{x}\right\}}_{j}^{2}\right)}$$

where the eigenvectors $\left\{\varphi_{a}\right\}$, extracted from the reference FE model, were compared with the reference eigenvectors $\left\{\varphi_{x}\right\}$, extracted from the bench test data. $N_{f}$ refers to the mode number, in ascending order.

The FRAC [20,21,22,23] is a frequency-dependent normalized single-value metric that estimates the correlation between two FRFs with the same excitation and response points. We computed it to assess the accuracy of the transfer functions found with the reference FE model. We contrasted the FRFs found from the reference FE model with those found in the bench test, restricting to the frequency range of concern, from 25 to 60 Hz, where the FRF peaks are most representative. We computed the FRAC as follows [20]:

(2) $$FRAC_{\left(a,x\right)}=\frac{{\left|\sum_{j=1}^{N_{f}}{(H_{a}\left(\omega_{j}\right)}^{H}.H_{x}\left(\omega_{j}\right))\right|}^{2}}{\left[\sum_{j=1}^{N_{f}}{(H_{a}\left(\omega_{j}\right)}^{H}.H_{a}\left(\omega_{j}\right))\right]\;\left[\sum_{j=1}^{N_{f}}{(H_{x}\left(\omega_{j}\right)}^{H}.H_{x}\left(\omega_{j}\right))\right]}$$

where the FRFs $H_{a}$, extracted from the reference FE model, were compared with the reference FRFs $H_{x}$, extracted from the bench test data. Both $H_{a}$ and $H_{x}$ are complex functions. The superscript H refers to the Hermitian, which is the transpose of the complex conjugate. $N_{f}$ and ω refer to the mode number and to the frequency value in ascending order.

#### 2.2.2. MAC Matrices

Table 2 gathers the MAC between the reference FE model of the vehicle body structure and the experimental test modes, the first obtained from the modal analysis simulation of the reference FE model and the second from the hammer impact bench test. From these results, we found that the main diagonal terms of the MAC matrix are higher than 0.9, which means that the modal results of the FEA are consistent with the experimental results [21].

We also noticed that the fifth and sixth modes are switched. We considered this switch to be an undesirable effect of the small number of accelerometers used in the bench test to acquire the local modes in the moonroof opening region. Even so, the main diagonal terms of the MAC matrix have high coefficients (>0.9) for all global modes. In any case, the effect of these switched modes affects only the moonroof area. Thus, we concluded that, despite this permutation of modes, the results are still consistent.

#### 2.2.3. FRAC Results

Table 3 shows the results of the FRAC analysis between the reference FE model and the experimental test of the vehicle body structure.

### 2.3. FE Test Models

#### 2.3.1. Model Definition

From the MAC and FRAC results, we detected a gap between the results obtained with the bench test and the reference FE model of the vehicle body structure. Since the reference FE model already includes all linear structural components and connections, we could not increase its accuracy just by considering the linear boundary conditions. Therefore, the FE model should also include other nonlinear elements. Since the contacts between the welded flanges of the vehicle body parts were present in the whole structure, we would also include them in the FE model of the body structure.

However, the change from linear to nonlinear boundary conditions, given by the addition of nonlinear contact elements on the whole structure, will exponentially increase the required calculation efforts. Therefore, it would be unfeasible to analyse this nonlinear FE model using the current computational resources available. 

**Figure 4:**
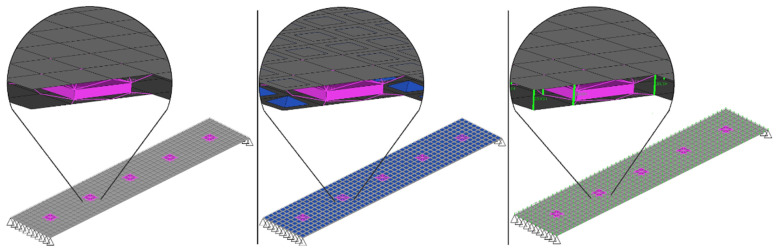
FE test models: without contact elements (**left**), with nonlinear contact elements (**center**) and with linear adjusted contact elements (**right**).

Taking into account this limitation, we built a linear FE test model (Figure 4, left), replicating at a small scale the design of the flanges of the vehicle body structure, according to the following parameters:

• Plate size [mm]: 250 × 50 × 1.0;
• Gap between upper and lower plates: 1.0 mm;
• Spot welds: 5 spots located every 50.0 mm; and
• System support condition: simply supported.

We also built a nonlinear FE test model (Figure 4, centre) by adding nonlinear contact elements to the linear FE test model in the surroundings of each spot weld. We used a contact static friction coefficient $\left\{\varphi_{S}\right\}=0.25$ [24,25,26]. We also built a third FE test model with linear contact elements (Figure 4, right), adjusting their stiffness and damping coefficients by comparing the results of the other two FE test models.

#### 2.3.2. Comparison of the First Eigenfrequencies

We performed a first set of analyses to assess the differences in the first eigenfrequency between the linear and nonlinear FE test models. To do this, we applied a load of 0.01 N to all nodes of the upper plate, exciting the system with a time-dependent sinusoidal enforced motion during 1 s, with a frequency ranging from 10 to 60 Hz, with a 1 Hz step.

Since the plate deflection shows a bending shape, we plotted the FFT of the deflection of the central node of the upper plate. From the FFT curves, we identified the first eigenfrequency of each model. 

**Figure 5:**
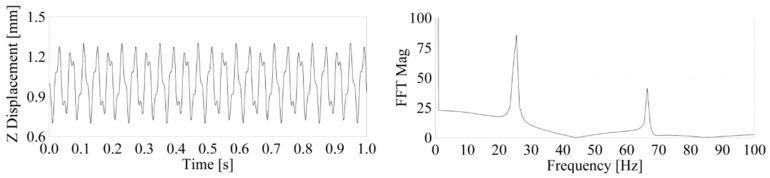
Linear FE model time response (**left**) and FFT response (**right**).

For the FE test model with only linear boundary conditions (Transient Direct solver solution), without contact representation, we found the first eigenfrequency at 66.4 Hz, with no variation of the results (<0.001 Hz) over the excitation frequency range (Figure 5).

**Figure 6:**
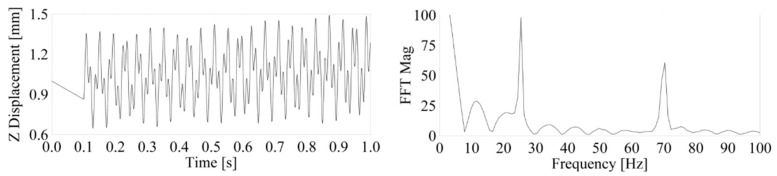
Nonlinear FE model time response (**left**) and time-to-frequency domain conversion of FFT response (**right**), at 25 Hz.

From the FE test model with nonlinear boundary conditions (Transient Non-Linear solver solution) and including nonlinear contacts, we found the first eigenfrequency at 70.3 Hz, with no other relevant results variation (<0.001 Hz) over the excitation frequency range (Figure 6). We used the difference between the first results of the two first FE test models (3.9 Hz) to adjust the stiffness of the spring-damper contact elements included in the third linear FE test model.

**Figure 7:**
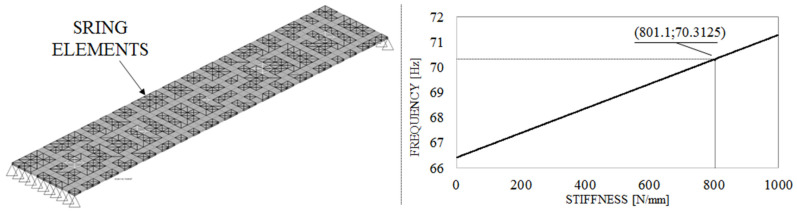
Adjusted linear FE reduced model (**left**) and spring element value interpolation (**right**).

Since the results for the first eigenfrequency remain constant over the frequency range of interest (from 10 to 60 Hz), we used a linear interpolation approach. We took the first eigenfrequency of the linear FE reduced model, $F_{a}=66.4\;Hz$ as a starting point. Since we did not include spring-damper elements in this model, we assumed its stiffness as $K_{a}=0.0\;N/\mathrm{mm}$. Next, we added spring-damper elements to the linear FE test model using an arbitrary stiffness value of $K_{b}=1000\;N/\mathrm{mm}$. We performed the same transient direct analyses as before and found the first eigenfrequency, $F_{b}$, at 71.3 Hz, with no results variation over the excitation frequency range. We then interpolated the first eigenfrequency of the nonlinear FE test model (70.3 Hz) between points *a* and *b* (Figure 7) and found the corresponding adjusted stiffness $K_{adj}=801.0\;N/mm$.

We also validated the adjusted linear spring-damper elements ($K_{adj}=801.0\;N/\mathrm{mm}$) by adding them to the linear FE test model. In this way, we found the first eigenfrequency at 71.3 Hz, the same value found with the nonlinear FE test model.

#### 2.3.3. Comparison of the Damping Decay Curves

**Figure 8:**
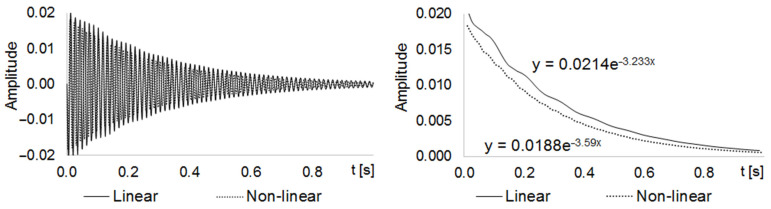
Linear and nonlinear z-dir displacement (**left**) and decaying rate (**right**) curves.

We performed a second set of analyses to assess the differences in the damping decay curves between the linear and nonlinear FE test models. To do this, we applied a pulse of 1 N on the central node of the upper plate for 0.01 s (Transient Modal solver solution). We took the time response of the node displacement over a 1 s span (Figure 8, left) and represented its envelope (Figure 8, right). In this way, we found the exponential damping decay curves for both the linear and nonlinear FE test models.

The exponents of the equations of these curves are their decaying rates, σ. We can also use this variable to calculate the damping ratio, ζ, of an undamped system [27], given by:

(3) $$\zeta =\frac{1}{\sqrt{1+{\left(\frac{2\pi }{\sigma }\right)}^{2}}}$$

being $\sigma ≜\ln\frac{y_{1}}{y_{2}}$ where $y_{1}$ and $y_{2}$ are the respective vibration amplitudes at two successive peaks of a decaying vibration, from which the decay rates $\sigma_{lin}=-3.233$ and $\sigma_{non-lin}=-3.590$ were calculated. It is also known that:

(4) $$\sigma =\frac{B}{2m}$$

From Equation (4), assuming that both linear and nonlinear systems have the same mass, we found that $B_{non-linear}=1.1104\cdot B_{linear}$. We then used this relationship to adjust the damping of the linear FE spring-damper elements, to simulate the effect of the nonlinear contacts.

**Figure 9:**
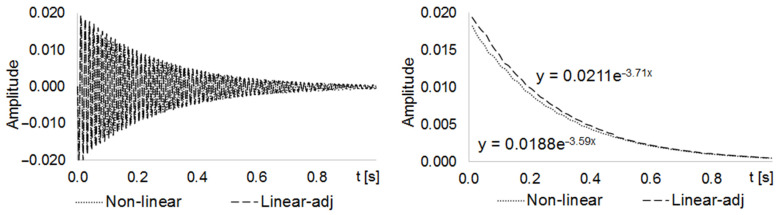
Nonlinear and linear adjusted z-dir displacement (**left**) and decaying rate (**right**) curves.

We also validated the adjusted linear spring-damper elements by adding them to the linear FE test model. Figure 9 shows the decay curves and equations found for the adjusted linear FE test model and the nonlinear FE test model.

Comparing the decay rate of the non-adjusted ($\sigma_{lin}=-3.233$) and adjusted ($\sigma_{adj}=-3.715)$ linear FE test models with the decay rate of the nonlinear FE test model $\left(\sigma_{non-lin}=-3.590\right)$, we found that the difference between the decay rates of the linear and nonlinear models decreased from 11.04% to 3.34% when using adjustment. This comparison can also be visually confirmed by comparing the curves and decay rates shown in Figure 8 and Figure 9.

## 3. Results

### 3.1. Adjusted FE Model of the Vehicle Body Structure

#### 3.1.1. Model Definition

**Figure 10:**
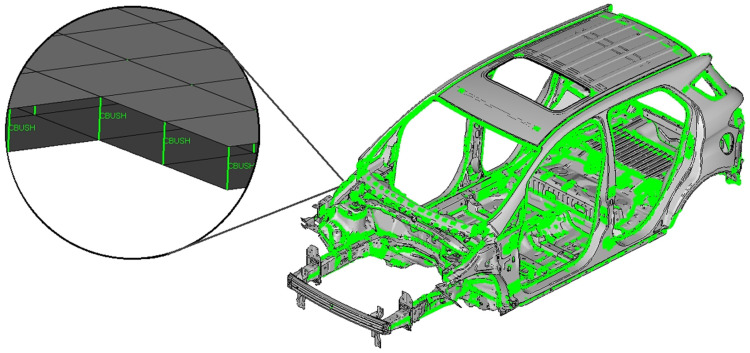
Adjusted linear FE model showing the added spring-damper elements.

We then built an adjusted linear FE model of the vehicle body structure (Figure 10). To this end, we added to the reference FE model of the vehicle body structure a set of adjusted spring-damper elements along all the welded flanges of the vehicle structure. For this purpose, we used the adjusted stiffness and damping values, $K_{adj}=801.0\;N/\mathrm{mm}$ and $B_{adj}=1.1104\;N.s/\mathrm{mm}$, previously calculated from the FE test models. From this adjusted FE model of the vehicle body structure, we obtained the same mode shapes and FRFs [15] as for the bench test and the reference FE model of the vehicle body structure.

Like for the bench test and the reference FE model of the vehicle body structure, we also simulated the impact hammer test from 0 to 100 Hz. To do so, we performed a frequency response analysis to capture the FRFs. We extracted the real part of the eigenvalues using the Lanczos’ method, considering the average structural damping ratio of 0.0044815, as found during the bench test.

**Figure 11:**
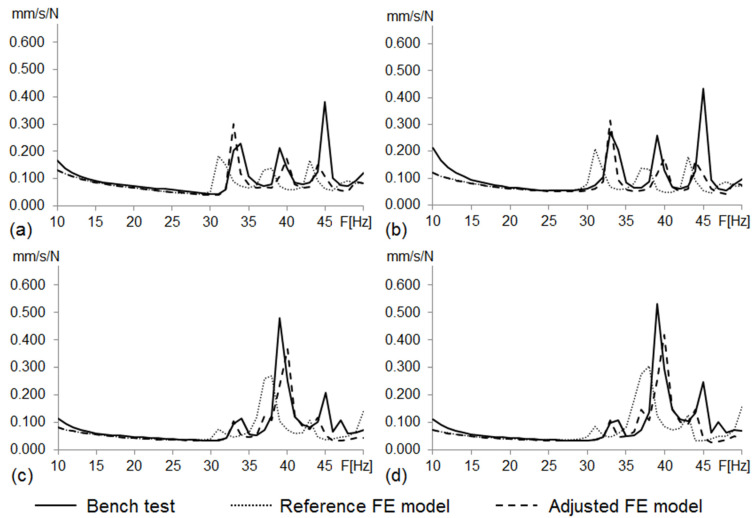
RMS curves from body structure bench test, reference and adjusted FE model FRFs (driver seat attachment vibration responses to unitary excitation). (**a**) Load at front left shock tower; (**b**) at front right shock tower; (**c**) at rear left shock tower; (**d**) at rear right shock tower.

Table 4 and Figure 11 show the eigenfrequencies and FRFs of the adjusted FE model of the vehicle body structure. They also gather those found from the bench test and the reference FE model of the vehicle body structure.

We assessed the accuracy of the new adjusted model using the MAC and FRAC metrics to compare the modal behaviour and the FRFs obtained with this model in comparison with the bench test.

Table 5 and Table 6 show the MAC and FRAC metrics of the adjusted FE model of the vehicle body structure, together with those of the reference FE model of the vehicle body structure. We calculated them by contrasting the results of the reference and adjusted FE models of the vehicle body structure with the bench test results.

#### 3.1.2. Eigenfrequencies

Table 4 shows the eigenfrequencies of these same mode shapes in both the reference and adjusted FE models of the vehicle body structure, together with those of the bench test.

#### 3.1.3. MAC Matrices

Table 5 gathers the MAC of the adjusted FE model of the vehicle body structure contrasted with the bench test. Despite the switch mentioned above between the fifth and sixth eigenmodes, the MAC coefficients are still higher than 0.9. Thus, the modal results also have consistent correspondence with the bench test data. The main diagonal terms on the MAC matrix of the adjusted FE model of the vehicle body structure are generally better (higher) than for the reference FE model, thus indicating an improved accuracy for the adjusted model.

#### 3.1.4. FRAC Coefficients

From the FRAC coefficients shown in Table 6, we computed the overall average FRAC for the adjusted FE model of the vehicle body structure, ${\mathrm{FRAC}}_{\mathrm{adj}}=0.4751$. It is 9.1% higher than the overall average FRAC for the reference FE model of the vehicle body structure, ${\mathrm{FRAC}}_{\mathrm{ref}}=0.4355$ (Table 3). This confirms the correlation improvement given by the proposed adjustment, also noticed by comparison of the MAC and the FRF plots.

#### 3.1.5. Frequency Response Functions (FRFs)

Figure 11 shows the root mean square (RMS) of the FRFs of the four excitation points for the bench test, the reference FE model of the vehicle body structure and the adjusted FE model of the vehicle body structure.

From these results, and based on the visual analysis of the plots, we found that the curves for the adjusted FE model generally fit better with the curves for the bench test than those for the reference FE model.

### 3.2. Optimization Analysis

We performed a weight optimization analysis of the vehicle body structure to evaluate the isolated effect of the proposed methodology. In this way, we could quantify the enhancement provided in terms of weight and cost savings. We considered the following restrictions to perform the optimization analysis:

- Disregard panels, since they were already set to the minimum gauge allowed;
- Disregard component brackets, since their main function is related to component tests;
- Avoid gauge changes smaller than 0.1 mm due to manufacturing restrictions;
- Avoid gauge changes higher than 15% of the initial gauge (if t < 1.0 mm) due to manufacturing restrictions for new tooling;
- Avoid gauge changes higher than 10% of the initial gauge (if t ≥ 1.0 mm) due to manufacturing restrictions for new tooling;
- Disregard critical parts related to frontal and lateral impact tests, since their main function is related to crash analysis; and
- Disregard critical parts related to chassis and powertrain attachments, since their main function is related to fatigue durability analysis.

**Figure 12:**
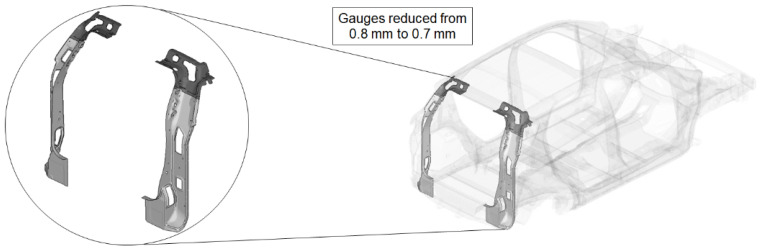
Optimized adjusted FE model of the vehicle body structure highlighting the parts with gauge reduction.

**Figure 13:**
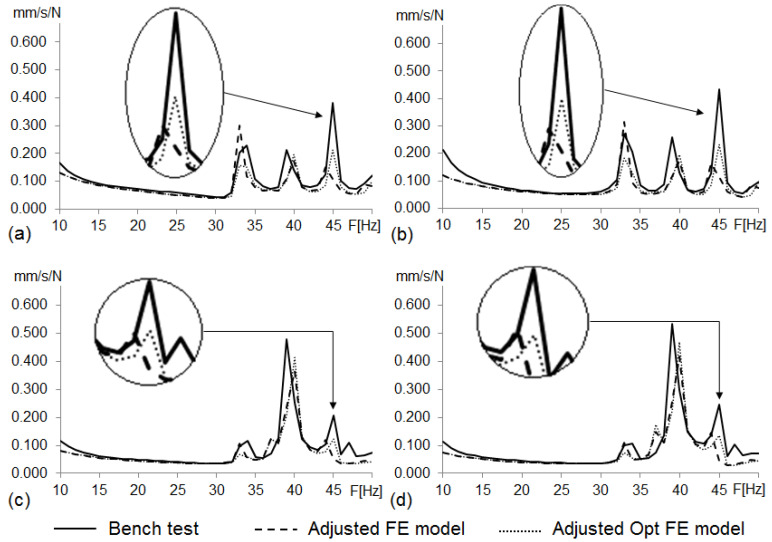
RMS curves from the body structure bench test, adjusted and optimized FE models FRFs (driver seat attachment vibration responses to unitary excitation). (**a**) Load at front left shock tower; (**b**) at front right shock tower; (**c**) at rear left shock tower; (**d**) at rear right shock tower.

The optimization analysis provided a gauge reduction in four parts of the vehicle body structure (Figure 12), resulting in a weight saving of 0.566 Kg. As expected, the FRF peaks of the optimized model moved in the direction of the bench test curves (Figure 13) but without exceeding their peaks.

**Figure 14:**
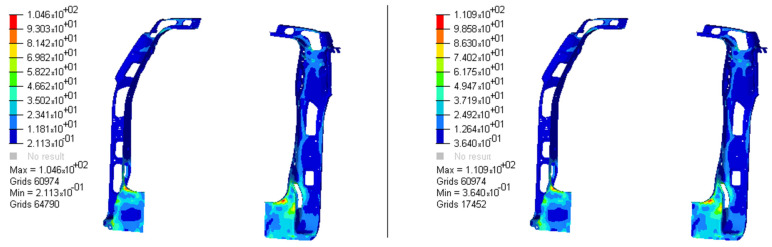
Maximum stress under torsional critical load on the adjusted FE model (**left**) and the adjusted optimized FE model (**right**).

In addition to this, we also analysed the stresses on the down-gauged components (0.7 mm thickness on the optimized adjusted FE model). We compared them with the stresses on the same components with the original gauge (0.8 mm thickness on the adjusted FE model). To this end, we performed a linear static calculation using an input torque of 3000 N.m applied in the front shock towers by opposite vertical loads. Based on the vehicle manufacturer’s experience in durability simulation test analysis, we partially constrained the rear shock towers to simulate a critical road test condition. We measured the maximum stress in the components considering the original and down-gauged conditions (Figure 14). Then we calculated the predicted lifetime [28] for each condition using the Neuber plasticity correction and the Smith–Topper–Watson mean stress correction, considering the properties of the SAE-1020 material. As a result, the original lifetime decreased from 5.681·10^6^ to 4.120·10^6^ cycles, but still complies with the material endurance limit [29].

The optimization analysis confirmed the improvement of the MAC and FRAC coefficients given by the adjusted model, thus corroborating the significant improvement of the correlation level provided by the proposed adjustment methodology.

To better illustrate the impact of the modelling approach proposed here, we also performed a weight-saving estimate as a result of this action, based on the following parameters:

• Sales in 2019 of the tested vehicle [30,31,32]: 282,371 units; and
• Weight savings per vehicle: 0.566 kg

As a result, we estimated material savings of 160 tons of steel per year for this vehicle model only. Considering that the life cycle of a vehicle can extend for approximately ten years [3] and the great diversity of vehicle models on the market, the total savings can reach very impactful numbers not only in economic terms, but also in environmental and social issues.

In this case, the reduction in the use of steel, which brings about important cost savings, also helps preserve natural resources. In addition, the weight reduction provided by this optimization improves the vehicle’s fuel economy performance, bringing another positive impact on the preservation of natural resources and the reduction of carbon emissions.

## 4. Discussion

Improving the correlation level between FE simulations and field tests is a key factor in improving the time- and cost-efficiency of many engineering projects. In particular, it is essential in the automotive industry. A possible solution to achieve this goal would be to add nonlinear elements and boundary conditions to traditional linear FE simulation models.

However, this change from linear to nonlinear conditions would substantially increase the computational time and resources required to perform an analysis. Therefore, when large structures are involved, such as in the case of vibration analysis of the vehicle body structure, the use of nonlinear models becomes an unfeasible alternative.

In this work, we propose a new methodology to increase the accuracy of linear models without compromising their complexity and feasibility. For this, we compared the MAC coefficients, the FRF curves and the FRAC coefficients obtained for both the reference and the adjusted FE models of the vehicle body structure, getting the following findings:

- Both the reference and the adjusted models have a consistent correspondence with respect to the bench test data (MAC > 0.9). The adjusted model shows slightly better MAC correspondence than the reference model;
- The FRF curve of the adjusted model fits better with the bench test results than the FRF curve of the reference model, especially around the 33 and 39 Hz peaks; and
- The overall average FRAC coefficient of the adjusted model (${\mathrm{FRAC}}_{\mathrm{adj}}=0.4751$) showed an improvement of 9.1% compared to the overall average FRAC coefficient of the reference model (${\mathrm{FRAC}}_{\mathrm{ref}}=0.4355$).

In addition, the optimization performed based on the results of the adjusted FE model generated a significant improvement in the weight of the vehicle body structure weight.

The analysis of these results confirmed the effectiveness of the proposed methodology. It fills the knowledge gap, initially identified, through a novel process for adjusting the stiffness and damping of spot-welded joints on thin-wall structures using small FE test models and including them into linear FE models of the entire vehicle body structure.

However, considering the results obtained, the following research opportunities to expand the correlation improvements achieved here can be further exploited:

- Improve the accuracy of the mesh representation of welding spots by proposing new element formulations;
- Improve the accuracy of the mesh representation of bolted connections by proposing new element formulations;
- Improve the accuracy of the mesh representation of adhesive connection joints by proposing new element formulations;
- Refine the representation of the FE model by incorporating variations in sheet metal thickness from stamping manufacturing processes;
- Improve the accuracy of experimental bench test results by proposing new acquisition strategies for raw data measurements; and
- Improve the accuracy of experimental bench test results by proposing new data processing methods for both vibration and damping evaluation.

These considerations, among others, will be introduced in future research models applied to vehicle structures for their refinement, either for vibration and noise analysis or even for durability and fatigue studies, aiming to increase the accuracy of their simulations and consequently improve the correlation level between the analytical and test results.

## Figures and Tables

**Table 1:** Eigenfrequencies and damping ratios of the vehicle body structure from the bench test.

Mode	Frequency [Hz]	Damping c/co [ ]	Mode Shape	Mode Picture
1	33.38	0.00513	Windshield Torsion	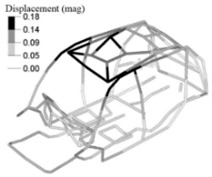
2	33.38	0.00390	1st Moonroof Bending	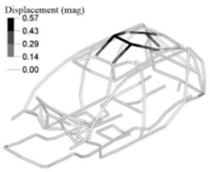
3	39.23	0.00567	Global Torsion	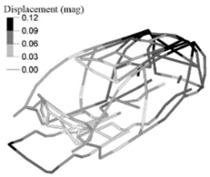
4	44.71	0.00228	Global Lateral Bending	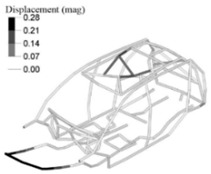
5	46.34	0.00642	2nd Moonroof Bending	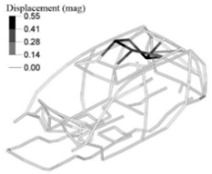
6	49.34	0.00344	Moonroof Torsion	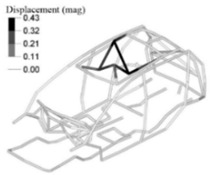
7	53.73	0.00453	Global Vertical Bending	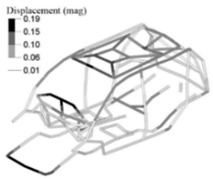

**Table 2:** MAC results for the reference FE vehicle body structure model.

-	-	BENCH TEST EIGENFREQUENCIES [Hz]						
-	-	33.4	33.4	39.2	44.7	46.3	49.3	53.7
REFERENCE FE MODEL EIGENFREQUENCIES [Hz]	31.3	0.97	0	0	0	0	0	0
	36.2	0.02	0.98	0	0	0.07	0	0
	37.5	0	0.01	0.98	0	0	0	0
	43.2	0	0	0.01	0.96	0	0.01	0
	47.3	0.01	0	0	0.04	0.01	0.91	0
	50.2	0	0.09	0	0	0.95	0.02	0
	53.0	0	0	0	0	0.06	0	0.93

**Table 3:** FRAC results for the reference FE model of the vehicle body structure.

Impact Point/Dir		FRAC	Impact Point/Dir		FRAC	Impact Point/Dir		FRAC
REFERENCE FE MODEL RESPONSE POINT: X-DIR	front left/x	0.9560	REFERENCE FE MODEL RESPONSE POINT: Y-DIR	front left/x	0.2315	REFERENCE FE MODEL RESPONSE POINT: Z-DIR	front left/x	0.8497
	front left/y	0.2270		front left/y	0.1654		front left/y	0.1428
	front left/z	0.4164		front left/z	0.2343		front left/z	0.6700
	front right/x	0.9320		front right/x	0.1547		front right/x	0.5928
	front right/y	0.1732		front right/y	0.1720		front right/y	0.1246
	front right/z	0.3994		front right/z	0.2403		front right/z	0.4808
	rear left/x	0.9647		rear left/x	0.2690		rear left/x	0.2206
	rear left/y	0.8365		rear left/y	0.2645		rear left/y	0.4020
	rear left/z	0.7581		rear left/z	0.1026		rear left/z	0.4220
	rear right/x	0.9682		rear right/x	0.2488		rear right/x	0.3059
	rear right/y	0.8887		rear right/y	0.2379		rear right/y	0.3238
	rear right/z	0.6374		rear right/z	0.1110		rear right/z	0.5542
Average x-dir		0.6798	Average y-dir		0.2027	Average z-dir		0.4241
-	-	-	-	-	-	Overall average		0.4355

**Table 4:** Eigenfrequencies of the bench test and the reference and adjusted FE models of the vehicle body structure.

Mode	Modal Shape	Bench Test [Hz]	Reference FE Model [Hz]	Adjusted FE Model [Hz]
1	Windshield Torsion	33.38	31.33	33.40
2	1st Moonroof Bending	33.38	36.22	37.10
3	Global Torsion	39.23	37.52	40.01
4	Global Lateral Bending	44.71	43.19	44.87
5	2nd Moonroof Bending	46.34	47.32	49.65
6	Moonroof Torsion	49.34	50.17	52.43
7	Global Vertical Bending	53.73	53.02	55.89

**Table 5:** MAC results of the adjusted FE model of the vehicle body structure.

-	-	BENCH TEST EIGENFREQUENCIES [Hz]						
-	-	33.4	33.4	39.2	44.7	46.3	49.3	53.7
REFERENCE FE MODELEIGENFREQUENCIES [Hz]	33.4	0.97	0	0	0	0	0	0
	37.1	0.02	0.99	0	0	0.10	0	0
	40.0	0	0	0.99	0	0	0	0
	44.9	0	0	0	0.98	0	0	0
	49.7	0.01	0	0	0.03	0.01	0.93	0
	52.4	0	0.07	0	0	0.97	0.02	0
	55.9	0	0	0	0	0.02	0	0.96

**Table 6:** FRAC results for the adjusted FE model of the vehicle body structure.

Impact Point/Dir		FRAC	Impact Point/Dir		FRAC	Impact Point/Dir		FRAC
REFERENCE FE MODEL RESPONSE POINT: X-DIR	front left/x	0.9666	REFERENCE FE MODEL RESPONSE POINT: Y-DIR	front left/x	0.1839	REFERENCE FE MODEL RESPONSE POINT: Z-DIR	front left/x	0.8323
	front left/y	0.1859		front left/y	0.1795		front left/y	0.1909
	front left/z	0.4848		front left/z	0.3990		front left/z	0.7250
	front right/x	0.9846		front right/x	0.1181		front right/x	0.5154
	front right/y	0.1774		front right/y	0.1946		front right/y	0.2316
	front right/z	0.4986		front right/z	0.3854		front right/z	0.4522
	rear left/x	0.9647		rear left/x	0.3333		rear left/x	0.2151
	rear left/y	0.8627		rear left/y	0.3187		rear left/y	0.3266
	rear left/z	0.8263		rear left/z	0.3838		rear left/z	0.3873
	rear right/x	0.9657		rear right/x	0.2916		rear right/x	0.3961
	rear right/y	0.9432		rear right/y	0.3388		rear right/y	0.2362
	rear right/z	0.6384		rear right/z	0.3813		rear right/z	0.5864
Average x-dir		0.7082	Average y-dir		0.2923	Average z-dir		0.4246
-	-	-	-	-	-	Overall average		0.4751

## Data Availability

Not applicable.
